# Screening of Cytotoxic *B. cereus* on Differentiated Caco-2 Cells and in Co-Culture with Mucus-Secreting (HT29-MTX) Cells

**DOI:** 10.3390/toxins8110320

**Published:** 2016-11-05

**Authors:** Virginie Castiaux, Laurie Laloux, Yves-Jacques Schneider, Jacques Mahillon

**Affiliations:** 1 Laboratory of Food and Environmental Microbiology, Earth and Life Institute, Université catholique de Louvain, Croix du Sud 2, L7.05.12, Louvain-la-Neuve B-1348, Belgium; virginie.castiaux@uclouvain.be; 2 Laboratory of Cellular, Nutritional and Toxicological Biochemistry, Institute of Life Sciences, Université catholique de Louvain, Croix du Sud 4-5, L7.07.03, Louvain-la-Neuve B-1348 , Belgium; laurie.laloux@uclouvain.be (L.L.); yjs@uclouvain.be (Y.-J.S.)

**Keywords:** *B. cereus*, enterotoxin, cytotoxicity, Caco-2, HT29-MTX

## Abstract

*B. cereus* is an opportunistic foodborne pathogen able to cause diarrhoea. However, the diarrhoeal potential of a *B. cereus* strain remains difficult to predict, because no simple correlation has yet been identified between the symptoms and a unique or a specific combination of virulence factors. In this study, 70 *B. cereus* strains with different origins (food poisonings, foods and environment) have been selected to assess their enterotoxicity. The *B. cereus* cell-free supernatants have been tested for their toxicity in vitro, on differentiated (21 day-old) Caco-2 cells, using their ATP content, LDH release and NR accumulation. The genetic determinants of the main potential enterotoxins and virulence factors (*ces*, *cytK*, *entFM*, *entS*, *hbl*, *nhe*, *nprA*, *piplC* and *sph*) have also been screened by PCR. This analysis showed that none of these genes was able to fully explain the enterotoxicity of *B. cereus* strains. Additionally, in order to assess a possible effect of the mucus layer in vitro, a cytotoxicity comparison between a monoculture (Caco-2 cells) and a co-culture (Caco-2 and HT29-MTX mucus-secreting cells) model has been performed with selected *B. cereus* supernatants. It appeared that, in these conditions, the mucus layer had no notable influence on the cytotoxicity of *B. cereus* supernatants.

## 1. Introduction

*B. cereus sensu lato* is a complex group of eight species genetically close but distinct in terms of pathogenicity. Indeed, this group comprises beneficial bacteria such as *Bacillus toyonensis*, used as probiotic or *Bacillus thuringiensis*, applied in agriculture as biopesticide, but also highly pathogenic species, like *Bacillus anthracis*, responsible for the anthrax disease. Moreover, some strains of *B. cereus sensu stricto* behave as opportunistic pathogens involved in non-gastrointestinal (e.g., endophtalmitis, periodontitis, meningitis or pneumonia) and gastrointestinal infections [1] with two types of symptoms: emetic or diarrhoeal.

The emetic syndrome is characterized by nausea and vomiting that occur 0.5 to 6 h after the meal. The symptoms are generally mild and disappear after less than 24 h, even though several complication cases have been reported [2,3,4,5,6]. The emetic syndrome is associated with a heat-stable toxin, the cereulide, which is generally preformed in food and resists to proteolysis and extreme pH [7]. The emesis seems to be due to the stimulation, by cereulide, of the efferent vagus nerve receptors (5-HT3) [8].

The diarrhoeal syndrome is characterized by abdominal pain, profuse diarrhoea with sometimes nausea. These symptoms occur 8 to 16 h after ingestion of *B. cereus* contaminated meal (infection dose: 10^5^–10^7^ CFU) and are probably due to the production of one or several enterotoxins by the *B. cereus* strains in the small intestine [9]. However, full light has not yet been shed on the exact molecules responsible for these symptoms. The most regularly cited enterotoxin candidates are: haemolysin BL (Hbl) [10], non-haemolytic enterotoxin (Nhe) [11] or cytotoxin K (CytK) [12]. Hbl is the only toxin for which a diarrhoeal activity was clearly demonstrated in vivo on rabbit intestines, using the purified toxin. Cytotoxic, haemolytic, vascular permeability and dermonecrotic activities of Hbl have also been demonstrated [13]. Nhe was first isolated from a foodborne outbreak in Norway [11]. A significant cytotoxicity effect of this toxin was observed on Vero and Caco-2 cells, although its haemolytic activity was limited [14,15]. Hbl and Nhe are tripartite enterotoxin complexes and belong to the α-helical pore-forming toxins family (α-PFT), like the Haemolysin E (HlyE) from *Escherichia coli* [15,16,17]. CytK is a single protein of 34 kDa belonging to the β-barrel pore-forming toxin family including other Gram-positive enterotoxins such as the α-haemolysin from *Staphylococcus aureus* or the β-toxin from *Clostridium perfringens* [12]. Two CytK variants have been described, CytK-1 and CytK-2. They display different pathogenicity with CytK-1 much more cytotoxic than CytK-2 on Vero and Caco-2 cells. This difference has been explained by a better conductance of the CytK-1-dependent channels than those from CytK-2 [18]. However, a strain (NVH 883/00) carrying the genetic determinants of CytK-1, but referenced as not toxic, has been found, which suggests that the virulence may also depend on the level of gene expression [19].

In addition to these three putative enterotoxins, other *B. cereus* haemolysins could participate to the gastrointestinal disease caused by *B. cereus* including: cereolysin O (CerO) [20], haemolysin II (HlyII) [21] and haemolysin III (HlyIII) [22]. Three phospholipases C have also been proposed to be involved in the *B. cereus* gastrointestinal syndrome: the phosphatidylinositol-specific PLC (PI-PLC), phosphatidylcholine PLC (PC-PLC) and the sphingomyelinase (Smase) [23,24]. Phopholipases are known to contribute to the pathogenesis of bacteria, through degradation of the mucus layer, destruction of the tissues or deregulation of cellular signalling cascades [25]. Therefore, it has been proposed that these molecules could act in concert with other enterotoxins to elicit diarrhoea [26]. Similarly, several studies have suggested that metalloproteases (e.g., InhA1, InhA2, InhA3 or NprA) could also contribute to the bacterial infection [27,28,29]. Finally, other compounds could also act as virulence factors contributing to the diarrhoeal pathotype of *B. cereus*, like the enterotoxin S (EntS) or FM (EntFM), described as cell-wall peptidase [30,31].

To date, no toxicological study has succeeded in demonstrating any correlation between one (or a combination) of these compounds and the diarrhoeal syndrome caused by *B. cereus*. This might potentially be due to the fact that previous cytotoxicity studies of *B. cereus* used CHO, Vero or Hep-2 cells. These cells lines are indeed very sensitive to toxins in general but do not necessarily reflect the specific interactions with the intestinal barrier and thereby with the diarrhoeal symptoms. Besides, several toxicological studies have been performed with human intestinal Caco-2 cells, but the cells were often not fully differentiated and therefore did not display the full characteristics of enterocytes [32]. This is of major importance though since it was demonstrated that the toxicological response between undifferentiated and differentiated Caco-2 cell cultures differs substantially [33]. Therefore, with the aim to better mimic the in vivo conditions of toxinogenesis, this study assessed the enterotoxicity of 70 *B. cereus* strains on fully differentiated Caco-2 cells. Additionally, this work also studied the interaction between the *B. cereus* supernatants and the mucus layer, using a co-culture (Caco-2/HT29-MTX) cell model [34].

## 2. Results

### 2.1. Cytotoxicity on Caco-2 Cell Monoculture

In order to assess the potential enterotoxicity of *B. cereus* strains, a panel of 70 isolates from different origins was selected. As indicated in Table 1, they originated from food poisoning (38 strains) and clinical cases (1), from food but not related to intoxications (15) and from the environment (13) or with an unknown origin (3). The cell-free supernatants of these *B. cereus* strains were tested by direct exposure to differentiated Caco-2 cell cultures, 21 days after seeding. After 3 h of exposure, the acute *B. cereus* cytotoxic effects were measured by two complementary methods: Lactate Dehydrogenase (LDH) release, which evaluates cell necrosis or post-apoptotic necrosis and Neutral Red (NR) uptake assays, which measures the cell capacity to acidify internal compartments (i.e., lysosomes and endosomes).

**Figure A1:**
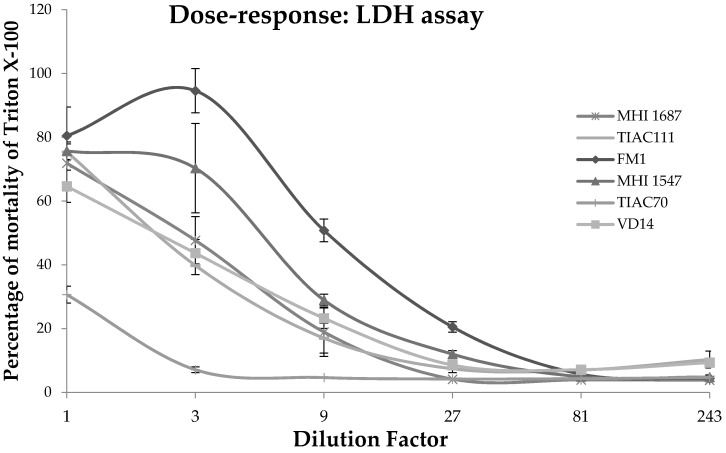
Dose-response effect on the cytotoxicity of *B. cereus* supernatants. The toxicity of six diluted *B. cereus* supernatants (MHI1687, TIAC111, FM1, MHI1547, TIAC 70 and VD14) was assessed on differentiated Caco-2 cells by LDH release assays. The supernatants were serial diluted (100%, 33%, 11%, 3.7%, 1.2% and 0.4% of supernatant) in HBSS. Similar results were obtained by NR uptake assays (data not shown).

Preliminary tests were performed to evaluate the toxicity of LB (Lysogenic Broth) medium used for the bacterial culture and confirmed that LB had no toxic effect on Caco-2 cells (See Table A1). Dilution assays of *B. cereus* supernatants were also conducted to determine the dilution factor that should be used for the subsequent analyses. These tests showed that the dose-responses were essentially strains-dependent and the diluted supernatants (dilution factor = 2) seemed to better discriminate the strains (Figure A1). Based on these results, it was decided that the supernatants would be diluted two times with HBSS buffer for all subsequent manipulations.

The Neutral Red uptake allowed to assess the cell capacity to acidify internal compartments and therefore to evaluate the viability of cells. Indeed NR uptake in the lysosomes occurs only in cells maintaining an active ATP-dependent proton pump. The results of this assay were thus expressed as percentages of Caco-2 cells viability over cells incubated with HBSS (100%) (negative control). The mean of the replicates (*N* = 3; *n* = 3) and standard deviation (SD) were calculated for each *B. cereus* supernatant (Table A1). Based on this test, a toxicity value (from 0 to 3) was arbitrarily attributed to each supernatant to facilitate the comparison with the LDH assay as detailed in the Materials and Methods section.

Cytotoxic effects of *B. cereus* supernatant have been also monitored by assaying the release of an intracellular enzyme by damaged cells in the culture medium, i.e., the Lactate Dehydrogenase (LDH). The results of this test were expressed in percentage of Caco-2 cell mortality over cells treated with Triton X-100 (positive cytotoxicity control) and the mean over the 9 measures (*N* = 3; *n* = 3) as well as their SD were calculated for each supernatant (Table A1). Similarly to NR assays, an arbitrary toxicity value (from 0 to 3) was attributed to each *B. cereus* supernatant (Table 1).

These two cytotoxicity assays were both used as cytotoxicity indicators although they do not monitor the same parameters. Indeed, the NR uptake by the lysosome is related to a healthy metabolic activity of cells, while LDH assay evaluates the cell membrane integrity, which is directly correlated to cell necrosis. However, these two complementary tests provided similar toxicity values, as shown in Table 1. Indeed the toxicity values obtained with LDH and NR assays were quite correlated (*x*^2^ = 107.46; *p* < 0.0001) and generally similar (for 54/70 strains). This correlation was also observed on the raw data (Table A1) and confirmed that the two tests were linearly correlated (*R^2^* = 0.88).

Nevertheless, for several strains with a low toxicity (A12, VD37, MHI13, MHI69, TIAC241, TIAC297 and TIAC67), it appeared that a harmful effect was only detected by the NR assays. In contrast, for more toxic supernatants (ATCC14579, MHI1494, B16, Bc1576, MHI1497 and NVH391-98), the maximum of toxicity value (3/3) was obtained with LDH dosage while a value of 2/3 was found with NR assays.

**Figure 1:**
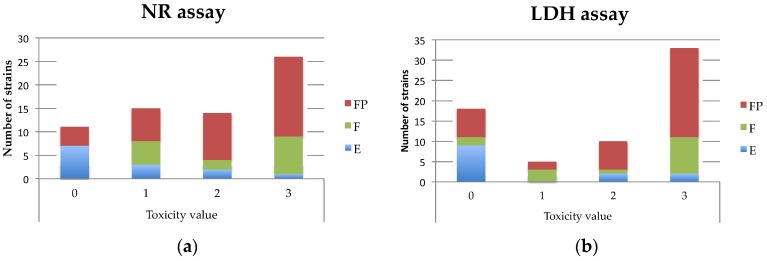
Distribution of *B. cereus* strains based on their origin (FP: food poisoning; F: food; E: Environment) and their toxicity values (arbitrary value from 0: none toxic to 3: highly cytotoxic). The histograms represent the strain distribution for each toxicity score based the NR (**a**) and on LDH (**b**) assays.

Concerning the distribution of the toxicity according to the strain origin (F, FP or E), the isolates from food poisonings were proportionnally more toxic than the environmental ones. As illustrated in Figure 1, based on the NR and LDH assays, the number of environmental strains was higher for the low toxicity values, while the opposite was observed for the food poisoning isolates. It should be noticed that the few clinical (1) and unknown (3) isolates were not included in Figure 1 and displayed high toxicity (values of 2 to 3).

### 2.2. Distribution of Potential Enterotoxin and Emetic Toxin Genetic Determinants in the B. cereus Collection

To better characterize the potential virulence of these *B. cereus* strains, the genetic determinants of the main putative enterotoxins and the emetic toxin were screened by PCR using the primers described in Table 2. The presence of the genetic determinants of 15 potential enterotoxins and virulence factors (cereulide, CytK, Hbl, HlyII, EntFM, EntS, Nhe, NprA, Smase, PI-PLC) were tested. As shown in Table 1, the strains possessed between 5 and 14 potential virulence genes. Among these genes, *entS*, *nheA* and *nprA* were present in all the strains tested. Previous studies already demonstrated the high prevalence of *nheA* and *nheB* in *B. cereus* strains [35,36,37,38]. Concerning *entS*, a lower occurrence rate was found by Minaard et al. (2007) (52%) and by Kim et al. (2015) in *B. cereus* (56%) and *B. thuringiensis* strains (62%) [39,40]. For NprA, a recent prevalence study has found that *nprA* was present in 52% of *B. cereus* strains [41] and Cadot et al. [28] showed that *nprA* was overexpressed in pathogenic strains. The present study also showed that *entFM*, *nheB*, *nheC*, *piplC* and *sph* were present in more than 90% of the strains, regardless their origin. Concerning the genetic determinants of Hbl, *hblA* was less frequent (50%) than *hblC* and *hblD*, which were respectively present in 57% and 54% of isolates (Table 1).

#### 2.2.1. Enterotoxin Genetic Determinant Profiles and Strain Origin

Concerning the number of virulence genes carried by each strain, the food poisoning (FP) isolates did not possess more potential enterotoxin genes than the other strains. Indeed, the proportion of strains containing at least 12 virulence genes was even higher in environmental (7/13; 54%) than in FP (18/38; 47%) isolates. The virulence genes were also generally widely distributed regardless the origin of strains, except for the emetic *ces* gene, only present in FP isolates and *sph*, which was always present in F and FP isolates, but not necessarily in E strains. However, for the *ces* genetic determinants, previous studies showed that cereulide-producing strains can be also found in F and E samples (e.g., [42,43]). Therefore, no clear correlation could be established between a specific virulence gene and the origin of a strain.

#### 2.2.2. Enterotoxin Genetic Determinant Profiles and Cytotoxicity

The potential relations between the presence of certain enterotoxin genes and the cytotoxicity against Caco-2 cells were assessed based on contingency tables analyses and chi-squared tests. Concerning the influence of the presence of *ces*, it appeared that several cereulide-producing strains, including emetic reference strains (F4810-72 [44] or H3081/97 [45]) had no cytotoxic effects on fully differentiated Caco-2 cells.

For the genetic determinants of Cytotoxin K, *cytK-2* was present in 64% of strains (45/70). Regarding the *cytK-1* form, only two positive strains were included in this study: NVH391-98 and NHV883-00. Fagerlund et al. demonstrated the high toxicity of NVH391-98 towards Vero cells while NVH883-00 was described as not toxic although it carried the *cytK-1* gene. However, in the present study, the cytotoxicity of NVH883-00 was assessed several times (*N* = 5, *n* = 3) and a certain toxicity was measured for several experiments but a lack of reproducibility was observed for this strain (See Table A1 and Table A2) in comparison with the others. This could potentially be due to a sensitive regulation of the toxin synthesis or their instability.

As mentioned in the previous section, *entFM* was present in most strains (66/70, 94%). Among the isolates that did not possess *entFM* (VD21, 45, NVH391-98 and TIAC896), there were both very toxic and not toxic strains. Similarly, the *entS* and *nprA* genes were detected in all isolates and did not seem to be essential factors in the *B. cereus* toxicity. The contingency table for *hlyII* also showed that this gene was as present in toxic than in safe strains. About *piplC* and *sph*, theses genes were only absent in non-toxic bacteria, but their presence did not guaranty the toxicity of a strain. These observations indicate that the corresponding molecules might potentially be involved in the pathogenicity, in combination with other toxic components.

Concerning *hbl* genes, their presence appeared not to be related to cytotoxicity. Indeed, among the strains that did not possess the three genetic determinants of the Hbl toxin, both highly toxic (ATCC10987, F5063/95, ISP3191, TIAC30, TIAC468, TIAC72, TIAC73) as well as non-toxic strains were found (e.g., MHI1698, V102 or VD21). Corollary, bacteria possessing the three *hbl* genes were cytotoxic but one strain (A1) displayed no toxicity on Caco-2 cells (Table 1).

To sum up, individually none of the tested genes was able to fully explain the virulence of *B. cereus* strains alone. Therefore, a multiple correspondence analysis (MCA) was also preformed on the data to assess a potential synergistic effect of different genes involved in the cytotoxicity, but no combination of genes could explain or correlate with the strain toxicity (data not shown).

### 2.3. Comparison of Cytotoxicity between Mono-(Caco-2 Cells) and Co-Cultures (Caco-2/HT29-MTX Cells)

In order to better mimic the in vivo conditions, an intestinal epithelium cell model composed of a mucus layer forming an unstirred water layer, seemed physiologically relevant. Therefore, the toxicity value obtained using a monoculture of enterocyte-like cells, i.e., Caco-2 cells, was compared to a co-culture of Caco-2 cells and HT29-MTX, the mucus-producing cells.

**Figure 2:**
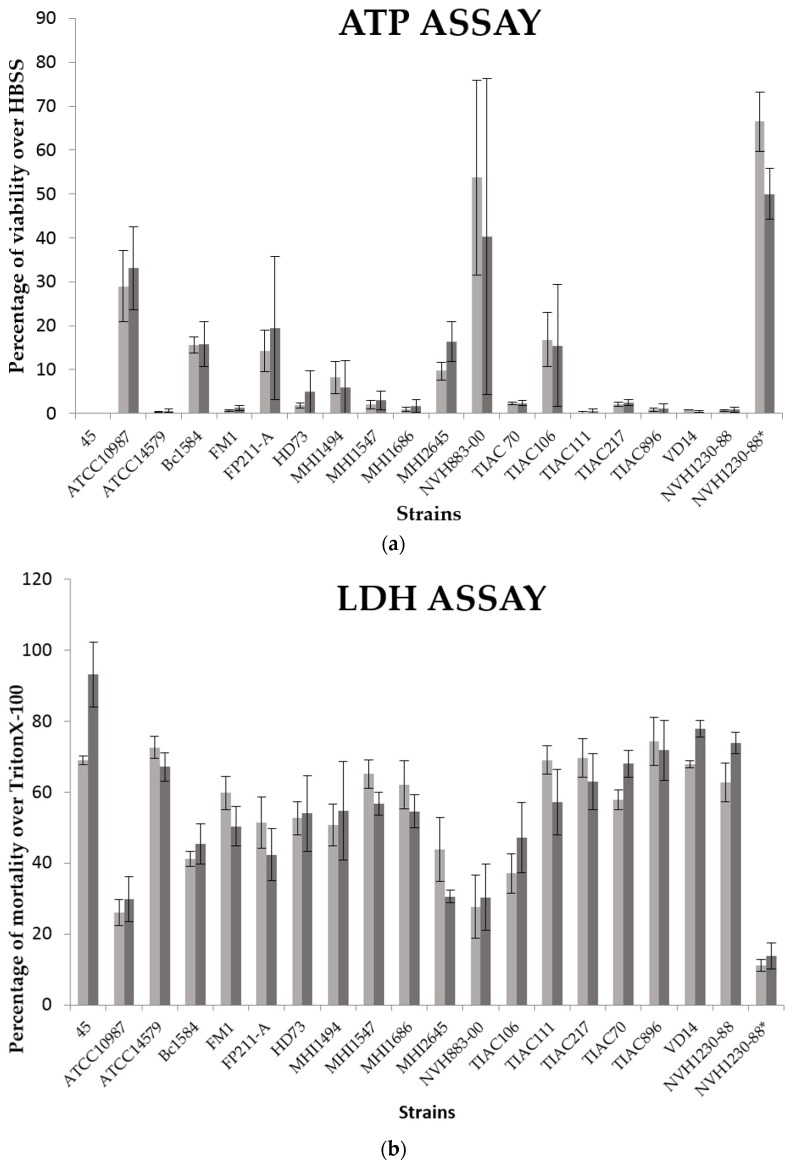
Comparison of cytotoxicity between the monoculture (Caco-2 cells) and the co-culture cellular model (Caco-2 and HT29-MTX cells). (**a**) ATP assay: the results are expressed in percentage of viability over cells incubated with HBSS; (**b**) LDH assay: the results are expressed in percentage of mortality over cells treated with Triton X-100. Monoculture results are represented in light grey while co-culture results are in dark grey. Percentage of viability (ATP) and mortality (LDH) are expressed as mean ± SEM (Standard deviation of the mean) (*n* = 6).

To compare the two in vitro cell models, 19 cytotoxic *B. cereus* supernatants were tested in parallel on mono- and co-culture cell models. The enterotoxicity of these supernatants was measured by LDH and ATP content assays. In comparison with the previous tests on monocultures, the ATP assay was chosen to replace the NR uptake assay that could potentially be affected by the presence of a protective mucus layer, which could decrease the diffusion rate of the hydrophobic neutral red molecule. The results were expressed in percentage of mortality over cells incubated with Triton X-100 (positive control) for LDH assays and in percentage of viability over cells incubated with HBSS (toxicity negative control) for ATP assays. For both cytotoxicity assays, the mean of results (*N* = 2; *n* = 3) and the standard deviations were calculated (Table A2) and are illustrated in Figure 2. Like for the previous tests, the NVH1230-88 (producing Nhe, HBL and CytK) supernatant was used as cytotoxic reference and the same supernatant, but inactivated by heat, was used as negative control.

The ATP assays confirmed the particularly high cytotoxicity of several strains (45, ATCC14579, FM-1, TIAC111, TIAC 217, TIAC 896, VD14 and NVH1230-88) (Figure 2A). Indeed, based on this assay, less than 5% of the cells remained unaffected after 3 h of exposure to these *B. cereus* supernatants. It also appeared that strain NVH883-00 was again particularly inconstant between both repetitions, as already shown in the previous test.

Concerning the comparison between the monoculture (Caco-2 cells) and the co-culture (Caco-2/HT29-MTX cells), on based on the LDH assays (Figure 2B and Table A2), VD14 is the only strain for which the toxicity values obtained by ATP and LDH assays were significantly higher on co-culture (Caco-2 with HT29-MTX cells) than in monoculture. For the other strains, no noticeable differences were observed between the two cell models. Therefore, the mucus layer did not seem to have a notable incidence on the *B. cereus* supernatant enterotoxicity.

## 3. Discussion

### 3.1. Toxicity Prediction

The enterotoxicity of *B. cereus* is generally attributed to three major potential enterotoxin candidates, namely Hbl, Nhe and CytK. However, numerous studies have already demonstrated that Nhe, Hbl and CytK (separately or in combination) do not seem to fully explain the diarrhoeal syndrome related to *B. cereus* [46,47,48,49]. Indeed, although *nhe* and *hbl* genes detection in *B. cereus* is generally applied to assess the enterotoxic potential of strains, it has been shown that the gene presence does not guaranty the cytotoxicity of the bacteria.

The present study confirmed that the enterotoxicity of *B. cereus* cell-free supernatant cannot be correlated with the presence of these enterotoxin genetic determinants. Therefore, the involvement of other putative enterotoxins and virulence factors have been assessed, including EntFM, EntS, NprA, Smase and PI-PLC. It appeared that the genetic determinants of these molecules, respectively, *entFM*, *entS*, *nprA*, *sph* and *piplC* did not seem to be related to the enterotoxicity observed against Caco-2 cells, even if their involvement cannot be fully excluded, particularly for *sph* and *piplC* that were only absent in safe strains.

The presence of the virulence genes does not guaranty the production and secretion of the corresponding protein [50]. Therefore, the presence of Nhe and HBL toxins in the *B. cereus* supernatants was assessed using immunodetection kits, which detect the presence of NheB and HBL-L2 enterotoxin subunits. These results confirmed that neither the Nhe and Hbl toxin production nor the presence of their genetic determinants was directly related to enterotoxicity of *B. cereus* strains (data not shown).

To fully exclude the involvement of all the putative virulence factors described here, a complete analysis and characterization of each *B. cereus* secretome should be undertaken. However, this analysis should also take into account of the toxin stability after its secretion by the bacteria. Indeed, Gilois et al. have demonstrated that certain toxins, e.g., CytK or CerO, are particularly unstable and do not persist more than two hours in *B. cereus* culture supernatants [50]. This may influence the cytotoxicity assessment of pathogenic strains and potentially explain the variability of the toxicity response observed between the repetitions, despite the standardization of the supernatant production.

In a nutshell, the screening of virulence genes or the toxin detection does not allow predicting the enteropathogenicity of isolates until now. This may be due to the fact that the cytotoxicity is modulated by a complex network of enterotoxin transcription regulators, which most likely integrates the environmental and nutritional status of the bacteria. Another possibility is that the molecule(s) responsible for the diarrhoeal syndrome has/have not been identified yet [47]. Therefore, a concerted approach of the *B. cereus* cytotoxicity in conjunction with kinetic transcriptomic and proteomic studies should be undertaken to identify the truly virulent factor(s) that cause(s) the *B. cereus* enterotoxicity.

### 3.2. Choice of Cellular Model to Assess Enteropathogenicity

To assess the enteropathogenic potential of *B. cereus* strains, Vero (monkey kidney cells) and Caco-2 (human colorectal adenocarcinoma) cell lines were preferentially selected. Vero cells present the advantage to be relatively easy to cultivate and very sensitive to a large panel of molecules [11]. However, Jeßberger et al. demonstrated that the sensitivity to enterotoxin is cell-line dependent [47]. With the aim to be as close as possible to in vivo conditions, Caco-2, human entero-epithelial cells were selected for this study because of their particularity to self-differentiate in enterocyte-like cells (e.g., polarization, tight junctions and protein expression) and mimic the intestinal epithelial barrier in in vitro cultures after 21 days cultivation. Nevertheless, in the vast majority of the cases, others toxicological studies on *B. cereus* were performed on Caco-2 cells that were not fully differentiated. This may lead to an overestimation of the cytotoxicity in comparison with a mature intestinal epithelium. Indeed, a mature Caco-2 cells monolayer could be more resistant to toxins than proliferating cells.

This hypothesis may explain why, in this cytotoxicological study, cereulide-producing strains displayed a very low toxicity against fully differentiated (21 days) Caco-2 cells while previous studies described emetic strains as cytotoxic vs. Caco-2 cells and various other cell lines [51,52]. Another explanation of these striking results may be the exposure duration used to assess the cytotoxicity. Indeed, the only study that also used 21 day-aged cells applied a confrontation time with cereulide of 3 days [52] while to reflect more the physiological reality, we decided to let the cells only 3 h in contact with the toxin. However, this specific cytotoxic response of emetic *B. cereus* should be further investigated, given that Guinebretière et al. (2010) have already described other emetic strains non (or weakly) cytotoxic on Caco-2 cells [53].

In order to be more physiologically relevant, a co-culture cellular model using Caco-2 and HT29-MTX mucin-producing cells was used. Indeed, it was demonstrated that the mucus layer (especially the inner, firmly adherent layer) may protect the epithelial barrier against certain macromolecules by size exclusion, hydrophobic interactions or due to its negative charges [54]. However, in this study, no significant difference in cytotoxicity level was observed between mono- and co-culture cell models.

Nevertheless, under in vivo conditions, bacteria and potentially toxins, generally penetrate the outer, loosely adherent layer [55]. This could play a protective effect against the degradation by proteases present in the lumen. Therefore it would be interesting to further investigate the mucus properties in terms of toxin protection and retention.

## 4. Materials and Methods

### 4.1. B. cereus Collection and DNA Extraction

70 *B. cereus* strains selected for this study were isolated from diverse origins: foods (15 strains), food-poisonings (38), environmental (13), clinical (1) or with unknown origin (3) as summarized in Table 1. These strains were collected within the framework of a Federal Public Service (FOD) Health, Food Chain Safety and Environment project (BACEREUS, RT09/2 project, Brussels, BE). The origin and the references related to these strains are described in Table A3. For all the experiments, the bacteria were cultured in Luria–Bertani medium (Oxoid, Dardilly, France) at 30 °C, under agitation (120 rpm).

The strain genomic DNA was extracted and purified using the DNeasy^®^ blood and tissue extraction kit (Qiagen, Hilden, Germany) with the recommended pre-treatment step for Gram-positive bacteria. In brief, 2 mL of an overnight culture of the bacteria in LB medium was centrifuged (10 min, 5000× *g*, 4 °C). The bacterial pellet was then resuspended in 180 μL of enzymatic lysis buffer (20 mM Tris-HCl, pH 8.0; 2 mM sodium EDTA; 1.2% Triton X-100; 20 mg/mL lysozyme) (Sigma-Aldrich, St. Louis, MO, USA) and incubated for 30 min at 37 °C. Then the extraction process was fully automated using the QIAcube System with the adequate protocol (Qiagen, Hilden, Germany). The purified DNA was resuspended in 150 μL of Buffer EA (10 mM Tris-HCl; 0.5 mM EDTA; pH 9.0) (Sigma-Aldrich, St. Louis, MO, USA) and stored at −20 °C for further use.

### 4.2. Detection of Virulence Factors and Enterotoxin Genes

The PCR screening of the genetic determinants of the main putative virulence factors involved in the *B. cereus* gastrointestinal toxi-infections was performed using the GoTaq^®^ Green Master Mix (Promega, Fitchburg, MA, USA). The different primers used are shown in Table 2. Thermal cycling parameters were 5 min at 94 °C, then 32 cycles of 1 min at 94 °C, 1 min at the annealing temperature (depending on the primer pair, see Table 2) and 1 min at 68 °C, and finally 10 min at 68 °C. The success of amplification was confirmed by gel electrophoresis at 100 V for 28 min on 0.8% agarose gel (Santa Cruz Biotechnology, Dallas, TX, USA) in Tris-Acetate-EDTA buffer (Sigma-Aldrich, St. Louis, MO, USA).

For several *B. cereus* strains, the presence of Nhe and HBL toxins in the supernatant was also assessed using the immunodetection kit Duopath Cereus Enterotoxins (Merck, Darmstadt, Germany).

### 4.3. Cell Line Cultures

Human colon carcinoma Caco-2 cells used to model the intestinal mucosa were provided by Dr. M. Rescigno (clone C2E; passage 10–30; University of Milano, Milan, Italy) and were seeded in 48-well plates (Corning-Costar, Corning, NY, USA) (6 × 10^4^ cells/well) pre-coated with type I collagen (Sigma-Aldrich, Saint-Louis, MO, USA) and incubated during 21 days after confluence. Cells were grown in Dulbecco’s modified Eagle’s minimal essential (DMEM) medium with 4.5 g/L glucose (Lonza, Basel, Switzerland). Media were supplemented with 10% (*v*/*v*) fetal bovine serum (FBS) (GE, Healthcare, Cramlington, UK), 1% (*v*/*v*) non-essential amino acids (Lonza, Basel, Switzerland), 1% (*v*/*v*) L-glutamine (Lonza, Basel, Switzerland) and 1% (*v*/*v*) penicillin-streptomycin (Lonza, Basel, Switzerland). Caco-2 cells were cultured at 37 °C under 10% CO_2_ and water saturated atmosphere.

Co-cultures of Caco-2 and HT29-MTX cells were used to assess the protective effect of mucin secretion. HT29 cells adapted to methotrexate (MTX, 10^−5^ M) were obtained from Dr. T. Lesuffleur (INSERM U505, Villejuif, France) were used between passages 30–50 with a seeding ratio Caco-2/HT29-MTX of 3:1, as previously described by Nollevaux et al. (2006) [34]. Together with Caco-2 cells, they form a co-culture that reproduces the two main cellular types encountered in the human intestinal epithelium.

### 4.4. Cytotoxicity Assays

*B. cereus* cell-free supernatants were prepared from a 15 h liquid culture in Luria–Bertani broth medium (Oxoid, Dardilly, France) at 30 °C, under agitation (120 rpm). This culture was centrifuged (4 °C, 15 min, 9000× *g*) and then filtrated with a 0.22 µm porosity filter. Strain NVH1230-88, known to produce at least the Nhe, Hbl and CytK toxins, was used as positive control. The negative control consisted on the same strain (NVH1230-88) for which the toxins were degraded by heat treatment (80 °C, 20 min).

After 21 days in 48-well plates, Caco-2 cells (or Caco-2 and HT-29 cells) were washed with phosphate buffered saline (PBS) (137 mM NaCl; 2.68 mM KCl; 1.14 mM KH_2_PO_4_; 8 mM Na_2_HPO_4_.2H_2_O; pH 7.2) (Sigma-Aldrich, St. Louis, MO, USA), incubated during 3 h with the *B. cereus* supernatants, diluted two times in HBSS buffer (Lonza, Basel, Switzerland) and then washed with PBS before assays.

To assess the cytotoxic effect of these supernatants several cytotoxicity assays were performed including ATP, NR and LDH assays. The first assesses the cell viability by the cytoplasmic ATP dosage. This quantification is based on the measurement of the luminescent signal generated by the conversion of luciferin by luciferase and the cytoplasmic ATP. The second assay determines the cell viability by the spectrophotometric determination of NR (3-amino-2-methyl-phenazine hydrochloride) taken up by viable cells and stored in their lysosomes [61]. The third test evaluates the lactate dehydrogenase (LDH) released by cells in necrosis [62]. For each experiment, several wells of each plate were dedicated to the positive (incubation with 1% of Triton X-100) (Sigma-Aldrich, St. Louis, MO, USA) and negative (incubation with Hank’s Balanced Salt Solution (HBSS), 100%) controls.

#### 4.4.1. Neutral Red Accumulation Assays

The treated cells (after washings) were incubated for 3 h with NR (750 µL at 0.33 mg/mL). Cells were then washed with PBS and NR was extracted using 300 µL of extracting solution (50% ethanol: 1% acetic acid) (Merck, Darmstadt, Germany). After 5–10 min of plate agitation the absorbance was measured by spectrophotometry at 540 nm [63]. The results were then expressed as percentages of Caco-2 cells viability over cells incubated with HBSS (toxicity negative control). The mean of the replicates and standard deviation (SD) were calculated for each *B. cereus* supernatant (Table A1 and Table A3). For the monoculture assays, with the aim to facilitate the comparison with the LDH assay, a toxicity value (from 0 to 3) was attributed to each supernatant as follows: 0 (non-cytotoxic), % of viability higher than 70%; 1, % of viability between 50% and 70%; 2, % of viability between 20% and 50%; 3, % of viability less than 20% (Table 1).

#### 4.4.2. LDH Release Assays

After the 3 h of exposure of the intestinal cells to the *B. cereus* supernatant, the lactate dehydrogenase activity released in the culture medium was assessed using the Cytotoxicity Detection Kit (LDH) (Roche, Basel, Switzerland). The liquid culture was centrifuged to remove the cell debris. Then, 1 µL of cell-free liquid culture was mixed with 99 µL of PBS in a 96 wells plate. 250 µL of Catalyst solution (containing diaphorase and NAD^+^) was mixed with 11, 25 mL of Dye solution (containing iodonitrotetrazolium and sodium lactate) and 100 µL of this reagent were added to each well. The reduction by the LDH enzyme of NAD^+^ to NADH was measured by spectrophotometry at 500 nm. The results of this test were expressed in percentage of Caco-2 cell mortality over cells treated with Triton X-100 (positive control). The mean over replicates as well as their SD were calculated for each supernatant (Table A1 and Table A3). Similarly to NR and for the Caco-2 monoculture assays, a toxicity value (from 0 to 3) was attributed to each *B. cereus* supernatant as follows: 0 (non-cytotoxic), % of mortality less than 20%; 1, % of mortality between 20% and 50%; 2, % of mortality between 50 and 70%; 3, % of mortality higher than 70% (Table 1).

#### 4.4.3. ATP Content Assays

The intracellular ATP content was monitored to assess the cytotoxicity of *B. cereus* supernatant for the co-culture assays instead of NR. Indeed, the mucin produced by the HT-29 cells could interfere with the NR uptake. The ATP content was measured using the luminescent CellTiter-Glo^®^ assay kit (Promega, Fitchburg, WI, USA). After the *B. cereus* supernatant exposition, the eukaryotic cells were rinsed twice with PBS. Then, 150 μL of HBSS were added to each well during 15 min at room temperature. The CellTiter-Glo^®^ buffer was mixed with the CellTiter-Glo^®^ substrate and 150 μL of this reagent mix was added to the wells. The plate was agitated during 2 min to homogenize the solution and accelerate cell lysis. In order to measure the highest signal possible, 200 μL of the cell solutions were transferred into a 96 wells white polystyrene plate, specific for luminescence measurements (Nunc™, Thermo Fisher Scientific, Waltham, MA, USA). The plate was shaken for 30 s and the luminescence recorded with a fluorimeter-luminometer (Fluoroskan Ascent™ FL, Thermo Fisher Scientific, Waltham, MA, USA) every 2 min during 20 min (0.5 s of integration time). This kinetics allowed identifying the time lapse where the maximum of luminescence was observed. The results of this test were expressed in percentage of Caco-2 cell viability over cells incubated with HBSS (negative control).

### 4.5. Statistical Analysis

For the Caco-2 cells toxicity assays, all measures were made in triplicates and each experiment was independently repeated three times (*n* = 9). Concerning the cytotoxicity comparison between co- and monocultures, all measures were made in triplicates and each experiment was repeated twice (*n* = 6). The normal distribution of the values was verified. Finally, the results were expressed as mean ± SD (see Table A1 and Table A2) or ±SEM (Figure 2). All the statistical correlation analyses including the contingency tables, the chi-square values and the MCA (Multi correspondence analyses) were performed with JMP 12 software (SAS Institute, Cary, NC, USA) and R statistics.

## Figures and Tables

**Table 1:** Characteristics of the 70 *B. cereus* strains used for this study.

Origin ^1^	Strain	Enterotoxin Genes or Genetic Determinants of Virulence Factors Tested ^2^															Toxicity Values ^3^	
		*ces*	*cytK1*	*cytK2*	*entS*	*entFM*	*hblC*	*hblD*	*hblA*	*hlyII*	*nheA*	*nheB*	*nheC*	*nprA*	*piplC*	*sph*	LDH	NR
C	F4501-83	-	-	+	+	+	+	+	+	+	+	+	+	+	+	+	3	3
E	A1	-	-	+	+	+	+	+	+	+	+	+	+	+	+	-	0	0
	A12	-	-	-	+	+	-	-	-	+	+	+	+	+	+	+	0	1
	AH621	-	-	+	+	+	+	+	+	-	+	+	+	+	+	+	0	0
	AH676	-	-	-	+	+	-	-	-	-	+	+	+	+	-	+	0	0
	ATCC14579	-	-	+	+	+	+	+	-	+	+	+	+	+	+	+	3	2
	HD73	-	-	+	+	+	+	+	+	+	+	+	+	+	+	+	2	2
	VD102	-	-	+	+	+	-	-	-	-	+	+	+	+	-	+	0	0
	VD107	-	-	-	+	+	-	+	-	-	+	+	+	+	+	-	0	0
	VD14	-	-	-	+	+	+	-	+	-	+	+	+	+	+	+	3	3
	VD21	-	-	-	+	-	-	-	-	-	+	+	-	+	-	+	0	0
	VD37	-	-	+	+	+	+	+	+	-	+	+	+	+	+	+	0	1
	VD48	-	-	+	+	+	+	+	+	+	+	+	+	+	+	+	2	1
	VD78	-	-	-	+	+	+	+	+	-	+	+	-	+	-	-	0	0
F	45	-	-	+	+	-	+	-	+	-	+	+	+	+	+	+	3	3
	27409	-	-	+	+	+	-	-	-	-	+	+	+	+	+	+	2	2
	390-88	-	-	-	+	+	-	+	-	+	+	+	+	+	+	+	1	1
	ATCC10987	-	-	+	+	+	-	-	-	-	+	+	+	+	+	+	3	3
	I13-2	-	-	+	+	+	+	+	-	+	+	+	+	+	+	+	3	3
	I8-5	-	-	+	+	+	+	+	+	-	+	+	+	+	+	+	3	3
	ISP2954	-	-	+	+	+	+	+	+	-	+	+	+	+	+	+	1	1
	ISP3191	-	-	-	+	+	-	-	-	+	+	+	+	+	+	+	3	3
	MHI13	-	-	-	+	+	-	-	-	+	+	+	+	+	+	+	0	1
	MHI1494	-	-	-	+	+	-	-	-	+	+	+	+	+	+	+	3	2
	MHI1547	-	-	+	+	+	+	+	+	-	+	+	+	+	+	+	3	3
	MHI1686	-	-	+	+	+	-	-	-	-	+	+	+	+	+	+	3	3
	MHI2645	-	-	+	+	+	+	+	+	-	+	+	+	+	+	+	3	3
	MHI69	-	-	-	+	+	-	-	-	-	+	+	+	+	+	+	0	1
	NVH883-00	-	+	-	+	+	+	+	+	+	+	+	+	+	+	+	1	1
FP	NVH1230-88	-	-	+	+	+	+	+	+	+	+	+	+	+	+	+	3	3
	B16	-	-	+	+	+	+	+	+	-	+	+	+	+	+	+	3	2
	B7	-	-	-	+	+	+	+	-	-	+	+	+	+	+	+	3	3
	Bc1558	-	-	+	+	+	-	-	-	-	+	+	+	+	+	+	3	3
	Bc1576	+	-	-	+	+	-	-	-	-	+	+	+	+	+	+	3	2
	Bc1584	+	-	-	+	+	-	-	-	-	+	+	+	+	+	+	2	2
	F4810-72	+	-	-	+	+	-	-	-	-	+	+	+	+	+	+	0	0
	FM-1	-	-	+	+	+	+	+	+	+	+	+	+	+	+	+	3	3
	FP211-A	+	-	-	+	+	-	-	-	-	+	+	+	+	+	+	3	3
	H3081/97	+	-	-	+	+	-	-	-	-	+	+	+	+	+	+	0	0
	MHI1497	-	-	+	+	+	+	+	+	-	+	+	+	+	+	+	3	2
	MHI1698	-	-	-	+	+	-	-	-	-	+	+	+	+	-	+	0	0
	NVH391-98	-	+	-	+	-	-	-	-	-	+	-	-	+	+	+	3	2
	TIAC106	-	-	+	+	+	+	+	+	-	+	+	+	+	+	+	3	3
	TIAC108	-	-	+	+	+	-	-	-	-	+	+	+	+	+	+	3	3
	TIAC111	-	-	+	+	+	+	+	+	+	+	+	+	+	+	+	3	3
	TIAC132	-	-	+	+	+	+	+	+	-	+	+	+	+	+	+	3	3
	TIAC139	-	-	-	+	+	-	-	-	-	+	+	-	+	+	+	0	0
	TIAC179	-	-	+	+	+	+	+	+	+	+	+	+	+	+	+	2	2
	TIAC180	-	-	+	+	+	-	-	-	-	+	+	+	+	+	+	3	1
	TIAC182	-	-	+	+	+	+	+	+	-	+	+	+	+	+	+	3	3
	TIAC217	-	-	+	+	+	+	+	+	-	+	+	+	+	+	+	3	3
	TIAC219	+	-	+	+	+	+	+	+	+	+	+	+	+	+	+	3	3
	TIAC247	-	-	-	+	+	-	-	-	-	+	+	+	+	+	+	0	1
	TIAC297	-	-	+	+	+	+	-	-	-	+	+	+	+	+	+	0	1
	TIAC299	-	-	+	+	+	+	+	+	-	+	+	+	+	+	+	1	1
	TIAC30	+	-	+	+	+	-	-	-	-	+	+	+	+	+	+	3	3
	TIAC371	-	-	-	+	+	+	-	-	+	+	+	+	+	+	+	2	2
	TIAC468	-	-	-	+	+	-	-	-	-	+	+	+	+	+	+	3	3
	TIAC67	-	-	-	+	+	+	+	-	-	+	+	-	+	+	+	0	1
	TIAC70	-	-	+	+	+	+	+	+	+	+	+	+	+	+	+	3	3
	TIAC71	-	-	+	+	+	+	+	+	+	+	+	+	+	+	+	2	2
	TIAC72	-	-	+	+	+	-	-	-	-	+	+	+	+	+	+	3	3
	TIAC73	-	-	+	+	+	-	-	-	-	+	+	+	+	+	+	3	3
	TIAC75	-	-	+	+	+	+	+	+	-	+	+	+	+	+	+	2	2
	TIAC76	-	-	+	+	+	+	+	+	+	+	+	-	+	+	+	2	1
	TIAC78	-	-	+	+	+	+	+	+	+	+	+	+	+	+	+	1	1
	TIAC896	-	-	+	+	-	+	+	+	+	+	+	+	+	+	+	2	2
U	15	-	-	+	+	+	+	+	+	-	+	+	+	+	+	+	2	2
	ATCC10876	-	-	+	+	+	+	+	+	-	+	+	+	+	+	+	2	2
	F5063/95	+	-	+	+	+	-	-	-	-	+	+	+	+	+	+	3	3

^1^ The isolates were classified according to their origins: C, clinical; E, environmental; F, Food; FP, Food Poisoning; and U, strains with unknown origin. ^2^ The presence of the genetics determinants of the *B. cereus* virulence factors were tested by PCR using primers described in Table 2. ^3^ The toxicity values were defined as described in Section 2.1. The scale includes four toxicity levels: from 0 (non-toxic) to 3 (highly cytotoxic) as detailed in the Materials and Methods section.

**Table 2:** Primers used for the PCR screening of the *B. cereus* toxin genes.

Gene	Name	Tm (°C)	Size (bp)	Sequences	References
*Ces*	EM1F	60	635	GACAAGAGAAATTTCTACGAGCAAGTACAAT	[56]
	EM1R			GCAGCCTTCCAATTACTCCTTCTGCCACAGT	
*cytK-1*	*CK1F*	57	426	CAATTCCAGGGGCAAGTGTC	[57]
	*CK1R*			CCTCGTGCATCTGTTTCATGAG	
*cytK-2*	CK2F	57	585	CAATCCCTGGCGCTAGTGCA	[57]
	*CK2R*			GTGIAGCCTGGACGAAGTTGG	
*entFM*	ENT-A	52	1269	ATGAAAAAAGTAATTTGCAGG	[30]
	ENT-B			TTAGTATGCTTTTGTGTAACC	
*entS*	TY123 F	60	581	GGTTTAGCAGCAGCTTCTGTAGCTGGCG	[30]
	TY125 R			GTTTCGTTAGATACAGCAGAACCACC	
*hlyII*	Fhly-II	48	868	GATTCTAAAGGAACTGTAG	[28]
	Rhly-II			GGTTATCAAGAGTAACTTG	
*hblC*	HC F	58	740	GATACYAATGTGGCAACTGC	[58]
	HC R			TTGAGACTGCTCGYTAGTTG	
*hblD*	HD F	58	829	ACCGGTAACACTATTCATGC	[58]
	HD R			GAGTCCATATGCTTAGATGC	
*hblA*	HA F	56	1154	AAGCAATGGAATACAATGGG	[58]
	HA R			AGAATCTAAATCATGCCACTGC	
*nheA*	NA F	56	755	GTTAGGATCACAATCACCGC	[58]
	NA R			ACGAATGTAATTTGAGTCGC	
*nheB*	NB F	54	743	TTTAGTAGTGGATCTGTACGC	[58]
	NB R			TTAATGTTCGTTAATCCTGC	
*nheC*	NC F	54	683	TGGATTCCAAGATGTAACG	[58]
	NC R			ATTACGACTTCTGCTTGTGC	
*nprA*	F-nprA-d	55	263	GTATACGGAGATGGTGATGG	[28]
	R-nprA-d			GGATCACTCATAGAGCGAAG	
*piplC*	PC105F	57	569	CGCTATCAATGGACCATGG	[59]
	PC106 R			GGACTATTCCATGCTGTACC	
*Sph*	Ph1	58	558	CGTGCCGATTTAATTGGGGC	[60]
	Ph2			CAATGTTTTAAACATGGATGCG	

## Abbreviations

The following abbreviations are used in this manuscript:

ATP	Adenosine triphosphate
C	Clinical isolates
Caco-2	Human epithelial colorectal adenocarcinoma cells
*ces*	Cereulide genetic determinants
CerO	Cereolysin O
CFU	Colony Forming Unit
CHO	Chinese Hamster Ovary
CytK	Cytotoxin K
DMEM	Dubelcco’s Modified Eagle’s minimal essential medium
E	Environmental isolates
EntFM	Enterotoxin FM
EntS	Enterotoxin S
F	Food isolates
FP	Food Poisoning isolates
HBSS	Hank’s Balanced Salt Solution
HBL	Haemolysin BL
HlyII	Haemolysin II
HT29-MTX	HT29 cells were treated with methotrexate and able to produce mucin
kDA	kilo Daltons
LB	Lysogenic Broth
LDH	Lactate Dehydrogenase
MCA	Multiple Correspondence Analysis
Nhe	Non-haemolytic enterotoxin
NprA	Metalloprotease: bacillolysin (*nprA*: genetic determinants of NprA)
NR	Neutral Red
PBS	Phosphate Buffer Saline
PCR	Polymerase Chain Reaction
PI-PLC	Phosphatidylinositol Phospholipase C (*pipl-C*: genetic determinants of PI-PLC)
SD	Standard Deviation
SEM	Standard Error of the Mean
Smase	Sphingomyelinase (*sph*: genetic determinants of Smase)
U	isolates with unknown origin

## Appendixes

**Table A1:** Cytotoxicity of *B. cereus* cell-free supernatants on Caco-2 cells. This toxicity was assessed using two complementary tests: the NR and the LDH assays. For the former, the results are expressed in % neutral red uptake over cells treated with HBSS (negative control), while for the latter, the results are given in % of lactate release over cells incubated with Triton X-100.

Treatments	LDH Assay		NR Assay	
	% of Mortality over Triton X-100		% of Viability over HBSS	
	Mean	±SD	Mean	±SD
15	58.14	±18.21	36.69	±34.12
45	89.85	±0.39	2.61	±2.43
27409	67.51	±30.44	46.72	±39.54
390-88	46.26	±9.98	57.10	±12.94
A1	0.05	±1.26	84.56	±24.82
A12	21.09	±15.14	92.60	±20.74
AH621	4.38	±2.75	103.99	±18.93
AH676	8.43	±8.15	92.31	±17.34
ATCC10876	59.06	±42.11	34.48	±49.43
ATCC10987	79.17	±4.79	6.56	±11.03
ATCC14579	69.16	±8.85	1.59	±2.21
B16	64.88	±11.98	13.55	±11.53
B7	80.78	±13.21	1.71	±1.22
Bc1558	74.26	±10.96	2.16	±1.02
Bc1576	66.55	±15.58	12.02	±9.31
Bc1584	53.47	±12.23	35.81	±27.40
F4501-83	80.28	±13.88	1.32	±1.08
F4810-72	2.33	±0.48	98.14	±4.63
F5063/95	84.60	±9.99	11.79	±11.87
FM-1	90.68	±9.43	0.84	±0.79
FP211-A	84.04	±3.97	1.54	±1.23
H3081/91	5.07	±1.66	76.34	±26.38
HD73	61.96	±15.59	33.74	±34.40
I13-2	72.21	±10.60	2.17	±1.31
I8-5	80.81	±12.06	4.72	±9.17
ISP2954	43.98	±4.00	51.82	±11.63
ISP3191	76.64	±14.21	13.00	±15.67
MHI13	28.34	±11.33	71.17	±18.98
MHI1494	66.60	±30.73	16.67	±22.17
MHI1497	61.18	±13.58	10.85	±15.85
MHI1547	92.74	±14.85	7.25	±10.58
MHI1686	84.69	±6.17	2.29	±1.82
MHI1698	2.32	±1.46	92.54	±8.19
MHI2645	84.03	±5.92	1.94	±1.27
MHI69	24.79	±6.22	89.46	±8.93
NVH391-98	57.11	±13.49	5.09	±1.89
NVH883-00	42.59	±37.08	51.39	±41.88
TIAC106	86.59	±11.64	1.38	±1.54
TIAC108	71.72	±5.13	5.86	±11.44
TIAC111	86.85	±15.55	2.45	±1.48
TIAC132	75.37	±12.88	4.36	±4.07
TIAC139	2.25	±1.23	97.76	±8.09
TIAC179	63.24	±28.70	49.29	±36.63
TIAC180	41.55	±32.90	7.69	±12.26
TIAC182	80.20	±21.67	18.62	±23.40
TIAC217	75.18	±6.41	4.56	±4.21
TIAC219	77.62	±11.79	2.17	±1.77
TIAC247	32.42	±14.44	94.48	±19.98
TIAC297	36.44	±12.30	84.47	±16.95
TIAC299	37.99	±11.58	67.08	±25.43
TIAC30	76.23	±16.16	6.58	±7.96
TIAC371	57.63	±28.46	25.71	±28.22
TIAC468	87.47	±27.66	11.64	±13.58
TIAC67	31.77	±39.17	94.24	±19.45
TIAC70	84.83	±9.49	0.90	±0.69
TIAC71	65.19	±25.54	31.21	±33.68
TIAC72	79.57	±13.86	1.26	±0.76
TIAC73	79.73	±9.57	1.52	±0.90
TIAC75	52.42	±24.35	47.59	±40.58
TIAC76	47.30	±8.44	38.72	±18.19
TIAC78	37.24	±14.40	59.31	±35.38
TIAC896	67.79	±10.61	21.80	±11.95
VD102	2.92	±0.90	96.36	±4.86
VD107	4.89	±4.11	105.17	±16.05
VD14	83.92	±31.05	2.91	±1.58
VD21	13.50	±6.60	101.68	±10.11
VD37	32.55	±15.36	76.41	±19.75
VD48	48.81	±16.71	44.53	±20.15
VD78	11.10	±6.46	101.28	±11.32
1230-88 ^1^	70.53	±14.42	0.71	±0.63
1230-88 ^1,^*	1.49	±0.87	91.22	±16.26
LB ^1^	1.75	±0.31	100.60	±14.60

^1^ Several controls were performed. First, the toxicity of the LB medium used for the bacterial culture has not cytotoxic effects on Caco-2 cells. The strain 1230-88 was used as positive controls like in previous studies [64,65]. The same strain, but inactivated by a heat treatment (10 min at 80 °C) was used as negative control: 1230-88 *.

**Table A2:** Cytotoxicity of *B. cereus* cell-free supernatants on monoculture (Caco-2 cells) and co-culture (Caco-2 and HT29-MTX cells). This toxicity was assessed using two complementary tests: the ATP and the LDH assays. For the former, the results are expressed in % in ATP content over cells treated with HBSS (negative control). For the latter, the results are given in % of lactate release over cells incubated with Triton X-100.

Treatments	LDH Assay				ATP Assay			
	% of Mortality over Triton X-100				% of Viability over HBSS			
	Caco-2		Caco-2 + HT29		Caco-2		Caco-2 + HT29	
	Mean	±SD	Mean	±SD	Mean	±SD	Mean	±SD
45	69.01	±2.98	93.16	±22.36	0.11	±0.13	0.08	±0.08
ATCC10987	25.98	±9.06	29.86	±15.51	28.94	±19.82	33.11	±9.50
ATCC14579	72.63	±7.59	67.10	±9.80	0.37	±0.24	0.58	±0.40
Bc1584	41.18	±4.95	45.44	±13.73	15.62	±4.39	15.74	±5.14
FM1	59.79	±11.34	50.37	±13.58	0.65	±0.52	1.24	±0.53
FP211	51.35	±17.81	42.39	±18.11	14.31	±11.66	19.50	±16.26
HD73	52.66	±11.31	54.02	±26.04	1.81	±1.42	4.95	±4.80
MHI1494	50.74	±14.60	54.69	±34.00	8.19	±8.80	5.87	±6.26
MHI1547	65.12	±9.82	56.79	±7.86	2.00	±2.48	2.98	±2.13
MHI1686	62.11	±16.75	54.58	±11.38	0.98	±1.07	1.72	±1.44
MHI2645	43.85	±15.50	30.57	±3.12	9.72	±3.52	16.32	±4.51
NVH883-00	27.72	±21.58	30.38	±22.86	53.76	±54.37	40.25	±35.98
TIAC106	37.15	±13.63	47.23	±24.35	16.77	±15.12	15.48	±13.93
TIAC111	69.08	±9.64	57.20	±22.38	0.22	±0.36	0.49	±0.55
TIAC217	69.59	±13.33	62.97	±19.07	2.12	±1.01	2.47	±0.68
TIAC70	57.85	±6.88	68.00	±9.08	2.33	±0.54	2.33	±0.55
TIAC896	74.28	±16.66	71.80	±20.77	0.86	±0.77	1.21	±1.04
VD14	67.94	±2.43	77.85	±5.59	0.88	±0.07	0.31	±0.12
NVH1230-88 ^1^	62.72	±13.21	73.85	±7.55	0.71	±0.46	0.90	±0.59
NVH1230-88 ^1,^*	11.22	±3.98	13.82	±9.24	66.45	±16.72	50.02	±5.73

^1^ Several controls were performed: strain 1230-88 was used as positive control. The same strain, but inactivated by a heat treatment (10 min at 80 °C) was used as negative control: 1230-88 *.

**Table A3:** Origins and references of the 70 *B. cereus* strains used in this study.

Name	Type of Isolates	Country of Isolation	References
1230-88	FP	Norway	[48]
15	U	Norway	[48]
45	F	Norway	[48]
27409	F	Belgium	ILVO ^1^
390-88	F	Norway	[48]
A1	E	Antarctica	MIAE ^2^
A12	E	France	[43]
AH621	E	Norway	[66]
AH676	E	Norway	[66]
ATCC10876	U	Unknown	[48]
ATCC10987	Food	Canada	[48]
ATCC14579	E	USA	[48]
B16	FP	Canada	[48]
B7	FP	Canada	[48]
Bc1558	FP	Belgium	MIAE
Bc1576	FP	Belgium	MIAE
Bc1584	FP	Belgium	MIAE
F4501-83	C	Norway	[67]
F4810-72	FP	United Kingdom	[43]
F5063/95	U	United Kingdom	K. Grant ^3^
FM-1	FP	Japan	[30]
FP211-A	FP	Belgium	[43]
H3081/97	FP	USA	[43]
HD73	E	China	[68]
I13-2	Food	China	[69]
I8-5	Food	China	[69]
ISP2954	FP	Belgium	MIAE
ISP3191	FP	Belgium	MIAE
MHI13	Food	Germany	[35]
MHI1494	Food	Germany	[35]
MHI1497	FP	Germany	[35]
MHI1547	Food	Germany	[35]
MHI1686	Food	Germany	[35]
MHI1698	FP	Germany	[35]
MHI2645	Food	Germany	[35]
MHI69	Food	Germany	[35]
NVH391-98	FP	France	[12]
NVH883-00	F	France	[12]
TIAC106	FP	Belgium	IPH ^4^
TIAC108	FP	Belgium	IPH
TIAC111	FP	Belgium	IPH
TIAC132	FP	Belgium	IPH
TIAC139	FP	Belgium	IPH
TIAC179	FP	Belgium	[48]
TIAC180	FP	Belgium	[48]
TIAC182	FP	Belgium	IPH
TIAC217	FP	Belgium	IPH
TIAC219	FP	Belgium	IPH
TIAC247	FP	Belgium	IPH
TIAC297	FP	Belgium	[48]
TIAC299	FP	Belgium	IPH
TIAC30	FP	Belgium	IPH
TIAC371	FP	Belgium	IPH
TIAC468	FP	Belgium	[48]
TIAC67	FP	Belgium	IPH
TIAC70	FP	Belgium	[48]
TIAC71	FP	Belgium	[48]
TIAC72	FP	Belgium	IPH
TIAC73	FP	Belgium	[48]
TIAC75	FP	Belgium	[48]
TIAC76	FP	Belgium	IPH
TIAC78	FP	Belgium	[48]
TIAC896	FP	Belgium	IPH
VD102	E	Guadeloupe	MIAE
VD107	E	Guadeloupe	MIAE
VD14	E	Spain	MIAE
VD21	E	Belgium	MIAE
VD37	E	Belgium	MIAE
VD48	E	Greenland	MIAE
VD78	E	Greenland	MIAE

^1^ ILVO: Instituut voor Landbouw- en Visserijonderzoek, Gent, Belgium; ^2^ MIAE: Laboratory of Food and Environment Microbiology, Louvain-la-Neuve, Belgium; ^3^ Dr K. Grant. Head of Foodborne Pathogens Reference Unit, Laboratory of Gastrointestinal Pathogens, Health Protection Agency Centre for Infections, London, England; ^4^ IPH: Institute of Public Health, Brussels, Belgium.
